# Evolution of Brain Morphology in Spontaneously Hypertensive and Wistar-Kyoto Rats From Early Adulthood to Aging: A Longitudinal Magnetic Resonance Imaging Study

**DOI:** 10.3389/fnagi.2021.757808

**Published:** 2021-11-30

**Authors:** Yingying Yang, Quan Zhang, Jialiang Ren, Qingfeng Zhu, Lixin Wang, Yongzhi Zhang, Zuojun Geng

**Affiliations:** ^1^Graduate School, Hebei Medical University, Shijiazhuang, China; ^2^Department of Imaging, The First Hospital of Qinhuangdao, Qinhuangdao, China; ^3^Tianjin Key Laboratory of Functional Imaging, Department of Radiology, Tianjin Medical University General Hospital, Tianjin, China; ^4^GE Healthcare China, Beijing, China; ^5^Department of Medical Imaging, The Second Hospital of Hebei Medical University, Shijiazhuang, China

**Keywords:** hypertension, aging, magnetic resonance imaging, voxel-based morphometry, brain atrophy, spontaneously hypertensive rat

## Abstract

The influence of hypertension and aging alone on brain structure has been described extensively. Our understanding of the interaction of hypertension with aging to brain morphology is still limited. We aimed to detect the synergistic effects of hypertension and aging on brain morphology and to describe the evolution patterns of cerebral atrophy from spatial and temporal perspectives. In 8 spontaneously hypertensive rats (SHRs) and 5 Wistar-Kyoto rats, high-resolution magnetic resonance imaging scans were longitudinally acquired at 10, 24, 52, and 80 weeks. We analyzed the tissue volumes of gray matter, white matter, cerebral spinal fluid, and total intracranial volume (TIV), and then evaluated gray matter volume in detail using voxel-based morphometry (VBM) and region of interest-based methods. There were interactive effects on hypertension and aging in tissue volumes of gray matter, white matter, and TIV, of which gray matter atrophy was most pronounced, especially in elderly SHRs. We identified the vulnerable gray matter volume with combined effects of hypertension and aging in the septal region, bilateral caudate putamen, hippocampus, primary somatosensory cortex, cerebellum, periaqueductal gray, right accumbens nucleus, and thalamus. We automatically extracted the septal region, anterior cingulate cortex, primary somatosensory cortex, caudate putamen, hippocampus, and accumbens nucleus and revealed an inverted-U trajectory of volume change in SHRs, with volume increase at the early phase and decline at the late phase. Hypertension interacts with aging to affect brain volume changes such as severe atrophy in elderly SHRs.

## Highlights

- Hypertension and aging have interactive effects on brain morphology.
- Longitudinal changes in gray matter volume are not uniform across space and time.
- Spontaneously hypertensive rats show an inverted-U trajectory of gray matter volume.
- MRI is a powerful tool for analyzing the dynamic evolution of whole brain morphology.

## Introduction

Hypertension was defined as 130 mmHg systolic or 80 mmHg diastolic blood pressure or greater according to the 2017 set of hypertension guidelines released by the American College of Cardiology and American Heart Association (Whelton et al., 2018). As the risk of hypertension increases with advancing age, its prevalence will increase dramatically with global aging. Additionally, an increasing number of younger individuals suffer from hypertension due to unhealthy lifestyles (Erdos et al., 2011; Mills et al., 2020). It has been well established that hypertension is an important risk factor for neuropathology. Multiple studies have demonstrated changes in cerebral functional integrity in hypertensive populations (Naumczyk et al., 2017; Feng et al., 2020), and brain structural deformities have also been observed, including hippocampal volume reductions and cortical atrophy (Korf et al., 2004; Gianaros et al., 2006). It is worth noting that brain atrophy also occurs in normal elderly individuals (Raz et al., 2005). However, the combined influence of hypertension and aging on brain morphology is not entirely clear. Previous clinical studies have shown that temporal and occipital regions appear most vulnerable due to the interactive effects of hypertension and age (Strassburger et al., 1997). Moreover, hypertension and aging may have strong interrelationship effects on brain damage, which is associated with cognitive decline (Kern et al., 2017).

It is difficult to investigate brain abnormalities in hypertensive populations while avoiding interferences from various environmental risk factors or treatment interventions. Furthermore, such clinical studies are typically cross-sectional, and only a few have attempted to longitudinally evaluate brain changes over a short age span (Gilsanz et al., 2017). Animal models provide convenience for exploring the impacts of hypertension on brain aging over the lifespan. Spontaneously hypertensive rats (SHRs), introduced by Okamoto and Aoki (1963), are the most extensively used animal model for essential hypertension. SHRs are normotensive at birth and progressively develop hypertension without any intervention. SHRs are commonly used to evaluate hypertensive brain damage and potential treatments (Chan et al., 2018; Shi et al., 2020). Postmortem histology analysis has revealed enlarged cerebral ventricles and reduced regional brain volumes in adult SHRs. Animal magnetic resonance imaging (MRI) is not only a powerful tool for whole-brain investigation but also a useful addition for noninvasively describing brain dynamic evolution. Brain atrophy is already present in SHRs at 7–9 weeks (Koundal et al., 2019). Although some aspects of cerebral damage in SHRs have been investigated, these results can differ since they are affected by aging (Li et al., 2016). Animal model experiments have shown that hypertension and aging induce an increase in ischemic susceptibility in aged SHRs (Lee et al., 2011). Research on cerebral blood volume with the combined effects of hypertension and aging suggests that a decrease in cerebral blood volume correlates with age but not hypertension, whereas a reduction in vasodilatory capacity is due to hypertension in SHRs based on near-infrared spectroscopy findings (Shaul et al., 2014). All these studies have added to our understanding about SHR brain aging; however, we could not accurately answer where and how hypertension exacerbates the brain morphological changes that accompany aging. Whether hypertension and aging affect only certain sensitive brain regions or a broader area remains unclear. SHR brain morphological trajectories with aging have never been depicted, and could present the evolution of brain atrophy as either on-going and progressive or relatively static. Hence, it is urgently needed to longitudinally characterize the spatial and temporal brain structural changes with brain aging in SHRs.

In the current study, we aimed to longitudinally assess brain morphology in SHRs and Wistar-Kyoto (WKY) rats from early adulthood to aging using *in vivo* MRI and to describe the evolution patterns of cerebral atrophy from spatial and temporal perspectives. We plan to delineate the combined effects of chronic hypertension on brain volume in the context of aging. We hypothesize that the combined effects of hypertension and aging would exacerbate cerebral atrophy. Cerebral morphological alterations are age dependent, and obvious brain atrophy may occur in aged SHRs. We intend to assess overall tissue volume changes in gray matter (GM), white matter (WM), and cerebral spinal fluid (CSF), and then evaluate regional GM morphological abnormalities in detail using voxel-based morphometry (VBM) and region of interest (ROI)-based methods. Quantitative analysis of brain volume alterations in SHRs over the life span will be necessary to understand the cumulative effects of hypertension on brain aging. These MRI markers of longitudinal changes in brain structure provide more comprehensive information about the evolution underlying the pathogenesis of chronic hypertension with brain aging.

## Materials and Methods

### Experimental Animals

Thirteen male SHRs and 10 WKY rats aged 8 weeks were purchased from Beijing Vital River Laboratory Animal Technology Company Limited. Five died in each of the two groups by the age of 80 weeks throughout their natural life cycle, so a total of 8 SHRs and 5 WKY rats were studied. All rats housed in an air-conditioned room (constant temperature 22–24°C, relative humidity 50–60%), at a light/dark cycle of 12 h. They were maintained on a standard pellet diet and tap water *ad libitum*. At the age of 20 weeks, blood pressure was measured by non-invasive blood pressure system. Body weight was recorded every week from 8 to 80 weeks. This study was approved by the Experimental Animal Ethics Committee of Hebei Medical University.

### Magnetic Resonance Imaging Scanning Protocol

MRI experiments were performed on two identical 7.0 T Bruker scanners (Pharma Scan 70/16 US) at the different sites. All rats were scanned 4 times repeatedly: at 10, 24, and 52 weeks at one place and subsequently 80 weeks at the other. Rats were initially anesthetized with 3% isoflurane in an induction chamber and then administered an intramuscular injection of 0.015 mg/kg dexmedetomidine into the back of the right thigh. Rats were placed in a prone position with a mixture of pure oxygen and isoflurane during MRI acquisition. The isoflurane level was adjusted between 0.5 and 1.2% to maintain breathing rate at 50–60 breath/min during scanning. A noninvasive pulse oximeter was attached to the left hind paw to ensure that oxygen saturation was above 96% during scanning. Body temperature was maintained at 37°C using a water circulation heating system. Whole brain T2-weighted MRI was acquired in coronal plane using a rapid acquisition with relaxation enhancement (RARE) sequence. Scan parameters: TR = 10,700 ms, effective TE = 36 ms, RARE factor = 8, FOV = 35 × 35 mm^2^, matrix size = 256 × 256, special resolution = 0.137 × 0.137 mm^2^, slice number = 90, slice thickness = 0.3 mm, number of averages = 4, and scan time = 22 min 50 s.

### Data Processing

We performed MRI data processing using the SPM12 toolbox in MATLAB (2013b). A whole brain population-specific template set for SHRs created by our team was used for image registration. First, all the T2-weighted images were multiplied by a factor of 10 to approximate the size of a human brain, which enabled the usage of data processing algorithms developed for humans. Second, these resized images were reoriented manually according to the template space. Third, the images were normalized and segmented based on our customized template set using the unified segmentation approach. In detail, the voxel values of the tissue maps were modulated by the Jacobian determinants of nonlinear components to account for the expansion or contraction in brain regions. Finally, the modulated GM volume images were smoothed by a 4 mm full width at half maximum Gaussian kernel for VBM. Individual GM, WM, and CSF volumes were calculated by multiplying total voxel numbers by mean volume values from modulated volume images. The total intracranial volume (TIV) was defined as the sum volume of GM, WM, and CSF. We also calculated the volume index of GM/TIV, WM/TIV, and CSF/TIV by dividing brain tissue volume by TIV in each rat. We automatically extracted certain ROIs from the modulation GM volume maps according to our template set, including the septal region, anterior cingulate cortex, primary somatosensory cortex, caudate putamen, hippocampus, and accumbens nucleus. The volume of each ROI was computed by multiplying the mean volume by the number of total voxels.

### Statistical Analysis

A flexible factorial design was performed within SPM12 for VBM analysis. We excluded voxels in which the volume value was below 0.2 in the smoothed GM volume images to ensure sufficient test effects. Voxel-level familywise error (FWE, *P* < 0.05) corrected for multiple comparisons with a minimal cluster size of 200 voxels was performed. Then, the mean value of each cluster was extracted to explore the *post hoc* analysis between groups at each time point using a *t*-test.

ROI-based volume changes over age between groups were evaluated with repeated-measures analysis of variance using SPSS (version 22.0), with group being the between-subject factor and age being the within-subject factor. Significant differences were examined using two sample *t*-tests on the volume at each time point to determine differences between groups and using paired *t*-tests in each group to determine the trend of volume changing with age. We correlated the TIV with the body weight in SHRs and WKY rats using Pearson’s correlative analysis. The threshold of statistical significance was *P* < 0.05.

## Results

### Brain Tissue Volume

**FIGURE 1:**
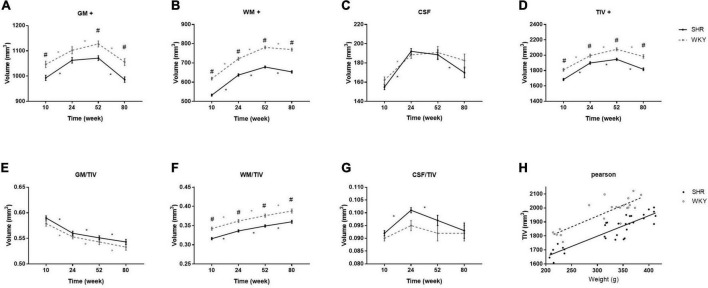
Comparisons of brain volume **(A–D)** and volume index **(E–G)** changes in SHRs and WKY rats at 10, 24, 52, and 80 weeks old. + indicates significant interactions of group and age. # Indicates a significant volume difference between SHRs and age-matched WKY rats, and * indicates a significant trend determined by paired *t*-tests in each group. Positive correlation between TIV and body weight in both groups **(H)**.

Figure 1 shows the brain volume and volume index changes in SHRs and WKY rats aged 10, 24, 52, and 80 weeks. There were interactions of group and age in the tissue volume of GM, WM, and TIV, and these tissue volumes were smaller in the SHRs than in WKY rats. Overall, the GM, WM, and TIV volumes exhibited continuous increases from 10 to 52 weeks but declined at different rates from 52 to 80 weeks in both groups. Compared with other tissues, GM volume demonstrated a steeper decline, especially in elderly SHRs. The temporal trajectories of the volume index showed that GM/TIV continuously declined and WM/TIV gradually increased in both groups. In addition, neither the CSF volume nor the CSF/TIV volume index differed between the two groups. Body weight was higher in the SHRs than in the WKY rats at 52 weeks old, while no difference was found at the other 3 time points. We observed a positive correlation between TIV and body weight in both groups: the correlation coefficients were 0.896 and 0.839 in SHRs and WKY rats, respectively. Table 1 shows the absolute volumes of GM, WM, CSF, and TIV in SHRs and WKY rats at 10, 24, 52, and 80 weeks old.

**TABLE 1:** Brain volume of the gray matter (GM), white matter (WM), cerebrospinal fluid (CSF), and total intracranial volume (TIV) in spontaneously hypertensive rats (SHRs) and Wistar-Kyoto (WKY) rats at 10, 24, 52, and 80 weeks old.

Tissue	Group	10 weeks	24 weeks	52 weeks	80 weeks
GM (mm^3^)	SHR	993.0 ± 10.2	1062.9 ± 11.0	1071.4 ± 9.3	986.2 ± 10.9
	WKY	1046.6 ± 12.8	1102.4 ± 13.9	1127.7 ± 11.7	1055.7 ± 13.7
WM (mm^3^)	SHR	532.6 ± 5.4	637.4 ± 6.2	679.0 ± 5.6	653.4 ± 6.1
	WKY	618.4 ± 6.8	721.7 ± 7.8	780.8 ± 7.1	769.6 ± 7.7
CSF (mm^3^)	SHR	154.9 ± 2.6	192.1 ± 2.9	188.5 ± 5.0	169.8 ± 5.2
	WKY	162.2 ± 3.3	188.4 ± 3.7	190.9 ± 6.3	182.6 ± 6.6
TIV (mm^3^)	SHR	1683.5 ± 13.9	1899.0 ± 15.9	1946.1 ± 15.4	1817.1 ± 18.3
	WKY	1809.4 ± 17.6	1994.5 ± 20.1	2077.0 ± 19.5	1983.3 ± 23.1

### Voxel-Based Gray Matter Volume

**FIGURE 2:**
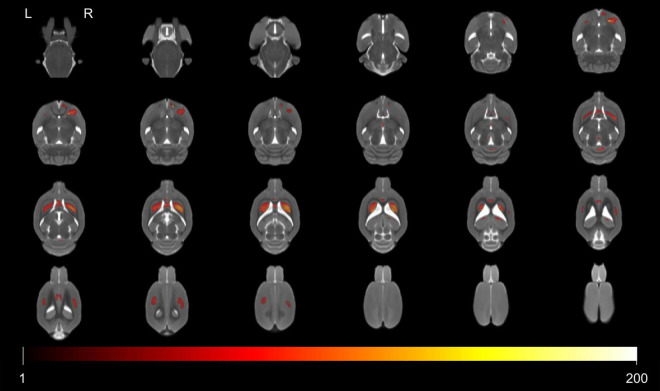
Colored voxels superimposed on the T2-weighted MRI template represent clusters with the interactions of group and age on gray matter volume (FWE, *P*-value < 0.05; threshold of 200 voxels). All 13 clusters included the septal region, bilateral caudate putamen, hippocampus, primary somatosensory cortex, cerebellum, periaqueductal gray, right accumbens nucleus, and thalamus. Note that L and R represent the left and right sides of the brain, respectively.

**FIGURE 3:**
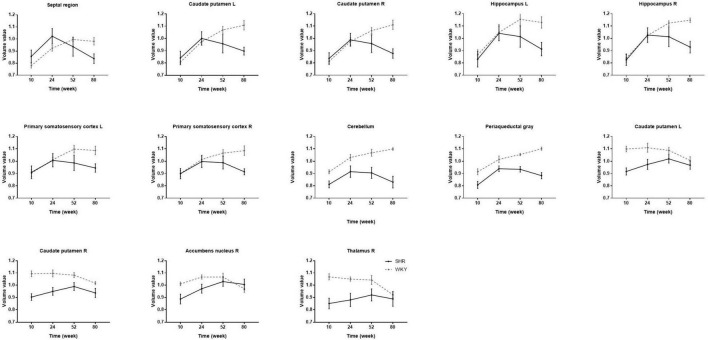
The volume changes in each cluster with interactions of group and age in SHRs and WKY rats aged 10, 24, 52, and 80 weeks. Temporal trajectories of each cluster morphology show obvious heterogeneity, which is different across regions. Compared with WKY rats, elderly SHRs exhibit severe gray matter atrophy. As early as 24 weeks of age, gray matter volume begins to atrophy significantly in the septal region, bilateral caudate putamen, and hippocampus.

The significant voxels were superimposed on the T2-weighted MRI template (FWE, *P* < 0.05; cluster extent > 200 voxels), which presented 13 clusters with interactions of group and age on the volume changes in SHRs and WKY rats (Figure 2). All these brain regions are summarized in Table 2. We performed a *post hoc* test by extracting the mean volume values from each cluster. Temporal trajectories of volume changes show obvious heterogeneity, differing across regions (Figure 3). In addition, elderly SHRs exhibit severe GM atrophy.

**TABLE 2:** Voxel-based morphometry analysis revealed some GM regions with interactions of group and age in SHRs and WKY rats.

Brain regions	Coordinates			Voxels	Peak F-score
	X	Y	Z		
Septal region	−4	−48	16	4,960	140.7
Caudate putamen L	−30	−35	−6	8,430	209.3
Caudate putamen R	31	−33	−7	9,483	204.1
Hippocampus L	−24	−30	−29	226	55.2
Hippocampus R	17	−32	−26	769	77.0
S1FL L	−36	−23	6	2,815	69.3
S1BF R, S1FL R	45	−26	0	3,333	66.0
Cerebellum	9	−37	−93	877	94.2
Periaqueductal gray	−4	−45	−62	239	91.1
Caudate putamen L	−32	−73	3	334	71.8
Caudate putamen R	29	−70	4	5,819	271.1
Accumbens nucleus R	18	−67	24	1,619	209.1
Thalamus R	0	−55	−32	321	92.4

*The names of these regions, the atlas coordinate of the peak point, the number of voxels, and the maximum F-score in the cluster are summarized in (Familywise error, P < 0.05; threshold of 200 voxels).*

*L, left; R, right; S1FL, Primary somatosensory cortex forelimb region; S1BF, Primary somatosensory cortex barrel field.*

### Region of Interest-Based Gray Matter Volume

**FIGURE 4:**
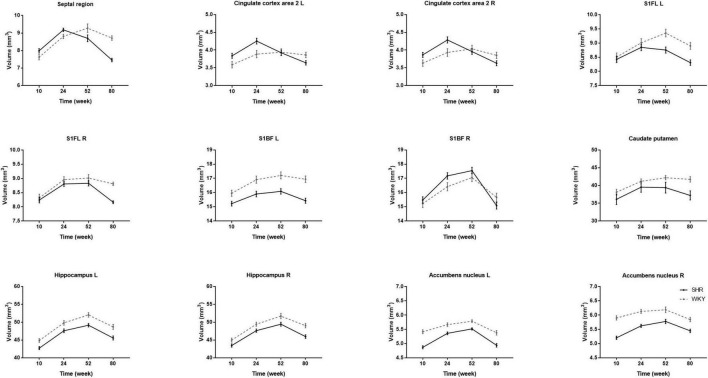
Comparisons of longitudinal changes in each ROI volume in SHRs and WKY rats at 10, 24, 52, and 80 weeks old. The caudate putamen volume is the average volume of both sides because there was no significant bilateral difference in the two groups at any time point. Temporal trajectories of gray matter volume show obvious heterogeneity between the two groups. The trajectories of the volume change show an inverted-U shape in SHRs, increasing at the early phase and declining at the late phase. Compared with WKY rats, regional unbalanced volume atrophy is more pronounced in SHRs. The rate of gray matter atrophy is fastest in the right primary somatosensory cortex barrel field in elderly SHRs.

We calculated the volume of the septal region, anterior cingulate cortex, primary somatosensory cortex, caudate putamen, hippocampus, and accumbens nucleus. There was no bilateral difference in caudate putamen volume in the two groups at all-time points, so the caudate putamen volume was represented as the average of both sides. The volume of other regions significantly differs between hemispheres at certain time points, so we analyzed the other regions on both sides. Table 3 presents regional GM volume in SHRs and WKY rats at different ages. Except for the hippocampus, the volume of other selected ROIs has interactions of group and age. Figure 4 demonstrates the GM volume longitudinal changes in both groups at 4 time points. Temporal trajectories of GM volume changes show obvious heterogeneity between the two groups. The trajectories of the volume changing with age show an inverted-U shape in SHRs, increasing at the early phase and declining at the late phase. Furthermore, unbalanced regional volume atrophy was more pronounced in the SHRs. The rate of GM atrophy was fastest in the right primary somatosensory cortex barrel field in elderly SHRs.

**TABLE 3:** Volume (mm^3^) of selected regions of interest of SHRs and WKY rats at different time points.

Region of interest	Group	10 weeks	24 weeks	52 weeks	80 weeks
Septal region	SHR	8.0 ± 0.1	9.2 ± 0.1	8.7 ± 0.2	7.5 ± 0.1
	WKY	7.6 ± 0.2	8.8 ± 0.1	9.3 ± 0.2	8.7 ± 0.1
Caudate putamen	SHR	36.1 ± 1.5	39.5 ± 1.4	39.4 ± 1.5	37.2 ± 1.3
	WKY	38.1 ± 0.8	41.2 ± 0.7	42.2 ± 0.6	41.7 ± 0.8
Hippocampus L	SHR	42.7 ± 0.4	47.6 ± 0.5	49.2 ± 0.5	45.6 ± 0.6
	WKY	44.9 ± 0.5	49.8 ± 0.7	52.0 ± 0.6	48.7 ± 0.7
Hippocampus R	SHR	43.5 ± 0.4	47.6 ± 0.5	49.5 ± 0.5	46.0 ± 0.5
	WKY	45.0 ± 0.6	49.5 ± 0.6	51.7 ± 0.7	49.1 ± 0.6
Accumbens nucleus L	SHR	4.9 ± 0.1	5.4 ± 0.1	5.5 ± 0.0	4.9 ± 0.1
	WKY	5.4 ± 0.1	5.7 ± 0.1	5.8 ± 0.0	5.4 ± 0.1
Accumbens nucleus R	SHR	5.2 ± 0.1	5.6 ± 0.1	5.8 ± 0.1	5.5 ± 0.1
	WKY	5.9 ± 0.1	6.1 ± 0.1	6.2 ± 0.1	5.8 ± 0.1

*There was no bilateral difference in caudate putamen volume in the two groups at all-time points, so the caudate putamen volume was represented as the average of both sides.*

## Discussion

To the best of our knowledge, this is the first longitudinal study combining hypertension with aging to detect the evolution of brain morphology in rats. Using high-resolution structural MRI, our study demonstrates a spatial and temporal pattern of brain volume alterations in SHRs and WKY rats from early adulthood to aging. The major strength of the current study lies in the longitudinal evaluation of the long-term changes in brain morphology. Our study produced two main findings. First, there are interactive effects of hypertension and aging on brain morphology: chronic hypertension makes cerebral atrophy more evident. Second, longitudinal changes in GM volume are not uniform, with different shrinkage magnitudes occurring across space and time.

### Brain Tissue Volume

The association of brain shrinkage with the interactions of hypertension and aging suggests that the effects of hypertension are not only cumulative but also progressive. In other words, the negative effects of chronic hypertension on cerebral atrophy become more evident with aging. Changes in brain tissue volume are not uniform. The volumes of GM, WM, and TIV were smaller in SHRs than in WKY rats. SHRs and WKY rats expressed similar brain tissue atrophy patterns but to different degrees. From 24 to 52 weeks, the GM volume in SHRs was relatively stable, while the GM volume in WKY rats continued to increase. Histopathological studies have previously reported GM volume loss in SHRs. It should be noted that *ex vivo* studies with brain fixation, extraction, and dehydration may result in ventricle collapse and anatomical shrinkage. An *in vivo* MRI study found that GM volume had no interaction effects between hypertension and aging in SHRs (Koundal et al., 2019). This controversy may be related to the age of the rats. Our study included elderly rats with chronic hypertension, while theirs was only based on early hypertensive rats. A clinical study revealed that cerebral perfusion increased with increasing blood pressure at low baseline but decreased at high baseline (Glodzik et al., 2019). Accordingly, we speculate that GM loss may be related to brain hypoperfusion caused by chronic hypertension.

We found that the volume index of GM/TIV gradually decreased, whereas the WM/TIV increased from 10 to 80 weeks in both groups. One preclinical study on hypertension and white matter disruption in inducible hypertensive rats reported that hypertension fails to disrupt white matter integrity in young or aged rats, which is consistent with our findings (Holland et al., 2015). Regional brain changes in aging adults with hypertension have confirmed white matter injury (Sabisz et al., 2019). We hypothesized that the opposite conclusion might be due to the lower proportion of white matter in rats. It is worth noting that the CSF volume shows nonsynchronous changes at the late phase, when it is reduced in SHRs and stable in WKY rats. These results do not seem to support the speculation that cerebral atrophy is compensated by an enlargement of the ventricles. Previous work confirmed that the CSF production rate and intracranial pressure are normal in SHRs. Perhaps we can explain the ventricle enlargement from the blood brain barrier permeability perspective. One study reported no evidence for blood brain barrier leakage in SHRs (Naessens et al., 2018); however, the opposing view was reported in aged SHRs (Wang et al., 2018). We believe that it is vital to explore the impacts of chronic hypertension on cerebral circulation (Cipolla et al., 2018). The body weight was higher in SHRs than in WKY rats at 52 weeks old, while there was no difference between groups at other time points. Linear regression analyses between body weight and TIV revealed that the correlation coefficients were similar between the two groups, and TIV in SHRs was consistently lower than that in WKY rats. Thus, we hypothesized that the smaller TIV in SHRs might be unrelated to their higher body weight.

### Voxel-Based Gray Matter Volume

In order to detect vulnerable GM volume alterations with interactive effects of hypertension and aging, we performed VBM analysis. We found that the interactive regions included certain cortical and subcortical regions such as the septal region, bilateral caudate putamen, hippocampus, primary somatosensory cortex, cerebellum, periaqueductal gray, right accumbens nucleus, and thalamus. *Post hoc* analysis revealed the patterns of GM volume changes. As early as 24 weeks of age, gray matter volume begins to atrophy obviously in certain brain regions, such as the septal region, bilateral caudate putamen, and hippocampus. These findings seem to contradict prior studies in aging SHRs showing that hippocampal volumes were similar in SHRs and WKY rats (Naessens et al., 2020). These conflicting results might be explained by the method of MRI data analysis and the rats’ age, since our studies used VBM analysis in rats with longer life span, while prior studies used manually drawn ROI-based methods in 10-month-old SHRs. Our study is generally consistent with previous clinical MRI studies that indicated that hypertension exacerbates the volume reductions accompanying advanced age (Strassburger et al., 1997). Our studies have shown that rats with chronic hypertension are much more prone to GM atrophy with aging in some specific brain regions.

### Region of Interest-Based Gray Matter Volume

The spatial heterogeneity of GM volume was detected using VBM analysis, while the temporal heterogeneity was explored using the ROI-based method. We used a longitudinal design to examine the evolution patterns of the GM volume over aging in rats with and without hypertension. Although longitudinal designs impede interindividual variation, one limitation is the shorter time windows (Elliott, 2020). We only selected 4 representative time points to obtain the trends in GM volume with age. We chose the septal region, anterior cingulate cortex, primary somatosensory cortex, caudate putamen, hippocampus, and accumbens nucleus as ROIs. We found that anterior cingulate cortex area 2, overlapping within the cluster of septal region, had the combined effects of hypertension and aging. Previous animal studies have also reported that anterior cingulate cortex damage is involved in hypertension-associated brain atrophy (Gianaros et al., 2006; Lai et al., 2021). We found that all the above ROIs had interactions, except for the bilateral hippocampus. We speculate that there should be hypertension-aging interactions in certain hippocampal subregions, but these interactions were offset by the relatively large volume of the hippocampus. A previous study observed an age-dependent neural reduction in the hippocampal CA1 area (Li et al., 2016). Our data suggested an inverted-U trajectory of GM volume change in SHR lifespan, with volume increase at the early phase and decline at the late phase. Therefore, the GM volume can rise, plateau, or decrease according to different time phases. One study showed that hippocampal volume increased with age in a normal aging rat strain (Alexander et al., 2020); however, another study found that hippocampal volume was similar in SHRs and WKY rats (Naessens et al., 2020). The difference between these results can be explained by our trajectory. Clinical studies reported that reduced hippocampal volume was correlated with hypertension duration and poorer cognitive aging (Triantafyllou et al., 2020; Van Etten et al., 2020). These results support our view that elderly SHRs experienced pronounced shrinkage. Moreover, the rate of GM atrophy was fastest in the right primary somatosensory cortex barrel field in elderly SHRs. We speculate that the right primary somatosensory cortex barrel field may be more sensitive to chronic hypoperfusion. Our results laterally support the neurovascular pathological theory with biphasic responses in cerebral blood flow and neurovascular coupling (Li et al., 2021).

### Magnetic Resonance Imaging Data Analysis

Structural MRI is a valid tool that can be used to noninvasively investigate alterations in the rat brain. We comprehensively assessed the spatial temporal course of GM volume change patterns using VBM and ROI-based methods. VBM analyzes the GM volume at the voxel level. A key advantage of VBM is that it allows for detecting whole brain volume automatically and objectively, while the disadvantage is that its accuracy might be impeded by registration errors. To minimize this problem, we used a custom template set for image registration. We checked the registration step by step and did not find any misregistration. Space smoothing can reduce image noise and enhance the statistical effect. We chose a 4 mm Gaussian smoothing kernel. A previous study confirmed that smoothing kernels did not significantly influence whole brain volume test-retest reliability in rats (Jing et al., 2018). Alternatively, we quantified regional GM volumes using automatic ROI-based analysis. Automatic ROI extraction omits manual drawing to improve the reliability of the results. Quantitative ROI-based analysis is beneficial for multicenter and cross-species comparisons.

### Animal Models

One benefit of animal models lies in the capability for within-subject longitudinal designs in the disease course. Many animal experiments on hypertensive brain damage have been performed in acute conditions (Meissner et al., 2017; Menard et al., 2018). The acute effects of hypertension on brain have been recognized for a long time (Iyonaga et al., 2019), while the long-term impacts of chronic hypertension on brain impairment remain incompletely understood. Only a few experiments have evaluated the influence of chronic hypertension (Willeman et al., 2019). SHRs are the most widely used animal model for human essential hypertension. SHRs are normotensive at birth and progressively develop hypertension without any intervening procedure. We chose scanning MRI at 10, 24, 52, and 80 weeks according to the features of SHRs. Blood pressure increases prominently at 3–10 weeks and remains stable for at least 20 weeks in SHRs. Animal models enable longitudinal design for analysis of chronic hypertension across the life cycle.

### Limitations

Several potential limitations should be noted. First, the MRI scanner at the last time point was not the same as before, although it was an identical type. To minimize the bias, we tried to keep the scanning protocol consistent. Second, the present work is based on male rats with a modest sample size. Strict-corrected statistical thresholds were restricted to minimize the risk of false positives. This is an issue of concern for higher blood pressure in male SHRs than in females (Amaral and Michelin, 2011). Few studies have explored sex differences in SHRs (Pietranera et al., 2016), and previous research investigations were mostly conducted in males. Further studies with larger sample sizes and longer follow-up periods are needed. Third, a longitudinal neuroimaging study of rats requires repeated anesthesia. A low dose of isoflurane in combination with dexmedetomidine is a viable option for longitudinal imaging in rats (Brynildsen et al., 2017). Fourth, as a longitudinal study covering natural aging rats, various comorbidities were inescapable, such as heart failure, atherosclerosis, and Alzheimer’s disease (Suzuki et al., 2015; Dinh et al., 2017; Chang et al., 2020). Since this phenomenon is common among elderly people, it may not prevent the clinical translation of our results. Finally, our present study primarily focused on evaluating brain morphology, and it would be interesting to compare the correlation of these volume results with cognition and behavioral function. Moreover, a recent clinical study confirmed that early onset hypertension was related to midlife cognitive function (Suvila et al., 2021). Future pathological- or molecular-level studies should expound the complex mechanisms of chronic hypertension related to brain aging.

## Conclusion

In conclusion, the current study presented a neuroimaging approach to longitudinally characterize brain morphology in SHRs and WKY rats from early adulthood to aging. There are interactive effects of hypertension and aging on brain volume alterations, and GM shrinkage is heterogeneous across space and time. Our results provide evidence supporting the notion that chronic hypertension accelerates brain aging. We hope that the longitudinal neuroimaging characteristics of aging SHRs may constitute a useful paradigm to explore the intricate pathological mechanisms of hypertension and aging.

## Data Availability Statement

The datasets presented in this article are not readily available because the data also form part of an ongoing study. Requests to access the datasets should be directed to YY.

## Ethics Statement

The animal study was reviewed and approved by the Laboratory Animal Ethical and Welfare Committee Hebei Medical University.

## Author Contributions

ZG, QZa, QZu, and YY conceived and designed the research. YY and YZ performed the experiment. YY and JR analyzed the data. YY and ZG wrote the manuscript. QZa, QZu, and LW participated in the discussion and provided the comments. All authors contributed to the article and approved the submitted version.

## Conflict of Interest

JR was employed by company GE Healthcare China. The remaining authors declare that the research was conducted in the absence of any commercial or financial relationships that could be construed as a potential conflict of interest.

## Publisher’s Note

All claims expressed in this article are solely those of the authors and do not necessarily represent those of their affiliated organizations, or those of the publisher, the editors and the reviewers. Any product that may be evaluated in this article, or claim that may be made by its manufacturer, is not guaranteed or endorsed by the publisher.

## References

[B1] Alexander G. E. Lin L. Yoshimaru E. S. Bharadwaj P. K. Bergfield K. L. Hoang L. T. (2020). Age-Related Regional Network Covariance of Magnetic Resonance Imaging Gray Matter in the Rat. *Front. Aging Neurosci.* 12:267. 10.3389/fnagi.2020.00267 33005147 PMC7479213

[B2] Amaral S. L. Michelin L. C. (2011). Effect of gender on training-induced vascular remodeling in SHR. *Braz. J. Med. Biol. Res.* 44 814–826. 10.1590/s0100-879x2011007500055 21537612

[B3] Brynildsen J. K. Hsu L. M. Ross T. J. Stein E. A. Yang Y. Lu H. (2017). Physiological characterization of a robust survival rodent fMRI method. *Magn. Reson. Imaging* 35 54–60. 10.1016/j.mri.2016.08.010 27580522

[B4] Chan S. L. Bishop N. Li Z. Cipolla M. J. (2018). Inhibition of PAI (Plasminogen Activator Inhibitor)-1 Improves Brain Collateral Perfusion and Injury After Acute Ischemic Stroke in Aged Hypertensive Rats. *Stroke* 49 1969–1976. 10.1161/STROKEAHA.118.022056 29991657 PMC6202199

[B5] Chang Y. M. Ashok Kumar K. Ju D. T. Ho T. J. Mahalakshmi B. Lin W. T. (2020). Dipeptide IF prevents the effects of hypertension-induced Alzheimer’s disease on long-term memory in the cortex of spontaneously hypertensive rats. *Environ. Toxicol.* 35 570–581. 10.1002/tox.22892 31889399

[B6] Cipolla M. J. Liebeskind D. S. Chan S. L. (2018). The importance of comorbidities in ischemic stroke: Impact of hypertension on the cerebral circulation. *J. Cereb. Blood Flow Metab.* 38 2129–2149. 10.1177/0271678X18800589 30198826 PMC6282213

[B7] Dinh Q. N. Chrissobolis S. Diep H. Chan C. T. Ferens D. Drummond G. R. (2017). Advanced atherosclerosis is associated with inflammation, vascular dysfunction and oxidative stress, but not hypertension. *Pharmacol. Res.* 116 70–76. 10.1016/j.phrs.2016.12.032 28017665

[B8] Elliott M. L. (2020). MRI-based biomarkers of accelerated aging and dementia risk in midlife: how close are we? *Ageing Res. Rev.* 61:101075. 10.1016/j.arr.2020.101075 32325150 PMC12430490

[B9] Erdos B. Kirichenko N. Whidden M. Basgut B. Woods M. Cudykier I. (2011). Effect of age on high-fat diet-induced hypertension. *Am. J. Physiol. Heart Circ. Physiol.* 301 H164–H172. 10.1152/ajpheart.01289.2010 21551274 PMC6189745

[B10] Feng R. Rolls E. T. Cheng W. Feng J. (2020). Hypertension is associated with reduced hippocampal connectivity and impaired memory. *EBioMedicine* 61:103082. 10.1016/j.ebiom.2020.103082 33132184 PMC7585137

[B11] Gianaros P. J. Greer P. J. Ryan C. M. Jennings J. R. (2006). Higher blood pressure predicts lower regional grey matter volume: Consequences on short-term information processing. *Neuroimage* 31 754–765. 10.1016/j.neuroimage.2006.01.003 16488626 PMC2254305

[B12] Gilsanz P. Mayeda E. R. Glymour M. M. Quesenberry C. P. Mungas D. M. DeCarli C. (2017). Female sex, early-onset hypertension, and risk of dementia. *Neurology* 89 1886–1893. 10.1212/WNL.0000000000004602 28978656 PMC5664296

[B13] Glodzik L. Rusinek H. Tsui W. Pirraglia E. Kim H. J. Deshpande A. (2019). Different Relationship Between Systolic Blood Pressure and Cerebral Perfusion in Subjects With and Without Hypertension. *Hypertension* 73 197–205. 10.1161/HYPERTENSIONAHA.118.11233 30571554 PMC7986962

[B14] Holland P. R. Pannozzo M. A. Bastin M. E. McNeilly A. D. Ferguson K. J. Caughey S. (2015). Hypertension fails to disrupt white matter integrity in young or aged Fisher (F44) Cyp1a1Ren2 transgenic rats. *J. Cereb. Blood Flow Metab.* 35 188–192. 10.1038/jcbfm.2014.201 25407269 PMC4426747

[B15] Iyonaga T. Shinohara K. Mastuura T. Hirooka Y. Tsutsui H. (2019). Brain perivascular macrophages contribute to the development of hypertension in stroke-prone spontaneously hypertensive rats *via* sympathetic activation. *Hypertens Res.* 43 99–110. 10.1038/s41440-019-0333-4 31541222

[B16] Jing B. Liu B. Li H. Lei J. Wang Z. Yang Y. (2018). Within-subject test-retest reliability of the atlas-based cortical volume measurement in the rat brain: A voxel-based morphometry study. *J. Neurosci, Methods* 307 46–52. 10.1016/j.jneumeth.2018.06.022 29960027 PMC6461491

[B17] Kern K. C. Wright C. B. Bergfield K. L. Fitzhugh M. C. Chen K. Moeller J. R. (2017). Blood Pressure Control in Aging Predicts Cerebral Atrophy Related to Small-Vessel White Matter Lesions. *Front. Aging Neurosci.* 9:132. 10.3389/fnagi.2017.00132 28555103 PMC5430031

[B18] Korf E. S. White L. R. Scheltens P. Launer L. J. (2004). Midlife blood pressure and the risk of hippocampal atrophy: the Honolulu Asia Aging Study. *Hypertension* 44 29–34. 10.1161/01.HYP.0000132475.32317.bb 15159381

[B19] Koundal S. Liu X. Sanggaard S. Mortensen K. Wardlaw J. Nedergaard M. (2019). Brain Morphometry and Longitudinal Relaxation Time of Spontaneously Hypertensive Rats (SHRs) in Early and Intermediate Stages of Hypertension Investigated by 3D VFA-SPGR MRI. *Neuroscience* 404 14–26. 10.1016/j.neuroscience.2019.01.030 30690138 PMC6450758

[B20] Lai A. Y. Joo I. L. Trivedi A. U. Dorr A. Hill M. E. Stefanovic B. (2021). Cerebrovascular damage after midlife transient hypertension in non-transgenic and Alzheimer’s disease rats. *Brain Res.* 1758:147369. 10.1016/j.brainres.2021.147369 33582120 PMC8005286

[B21] Lee T. H. Liu H. L. Yang S. T. Yang J. T. Yeh M. Y. Lin J. R. (2011). Effects of aging and hypertension on cerebral ischemic susceptibility: Evidenced by MR diffusion–perfusion study in rat. *Exp. Neurol.* 227 314–321. 10.1016/j.expneurol.2010.12.003 21146526

[B22] Li Y. Li R. Liu M. Nie Z. Muir E. R. Duong T. Q. (2021). MRI study of cerebral blood flow, vascular reactivity, and vascular coupling in systemic hypertension. *Brain Res.* 1753 147224. 10.1016/j.brainres.2020.147224 33358732

[B23] Li Y. Liu J. Gao D. Wei J. Yuan H. Niu X. (2016). Age-related changes in hypertensive brain damage in the hippocampi of spontaneously hypertensive rats. *Mol. Med. Rep.* 13 2552–2560. 10.3892/mmr.2016.4853 26846626 PMC4768967

[B24] Meissner A. Minnerup J. Soria G. Planas A. M. (2017). Structural and functional brain alterations in a murine model of Angiotensin II-induced hypertension. *J. Neurochem.* 140 509–521. 10.1111/jnc.13905 27874975

[B25] Menard B. Chazalviel L. Roussel S. Bernaudin M. Touzani O. (2018). Two-kidney one-clip is a pertinent approach to integrate arterial hypertension in animal models of stroke: Serial magnetic resonance imaging studies of brain lesions before and during cerebral ischemia. *J. Cereb. Blood Flow Metab.* 38 1769–1780. 10.1177/0271678X17715813 28617154 PMC6168912

[B26] Mills K. T. Stefanescu A. He J. (2020). The global epidemiology of hypertension. *Nat. Rev. Nephrol.* 16 223–237. 10.1038/s41581-019-0244-2 32024986 PMC7998524

[B27] Naessens D. M. P. Coolen B. F. de Vos J. VanBavel E. Strijkers G. J. Bakker E. (2020). Altered brain fluid management in a rat model of arterial hypertension. *Fluids Barriers CNS.* 17:41. 10.1186/s12987-020-00203-6 32590994 PMC7318739

[B28] Naessens D. M. P. de Vos J. VanBavel E. Bakker E. (2018). Blood-brain and blood-cerebrospinal fluid barrier permeability in spontaneously hypertensive rats. *Fluids Barriers CNS.* 15:26. 10.1186/s12987-018-0112-7 30244677 PMC6151927

[B29] Naumczyk P. Sabisz A. Witkowska M. Graff B. Jodzio K. Ga̧secki D. (2017). Compensatory functional reorganization may precede hypertension-related brain damage and cognitive decline. *J. Hypertens* 35 1252–1262. 10.1097/HJH.0000000000001293 28169883 PMC5404398

[B30] Okamoto K. Aoki K. (1963). Development of a strain of spontaneously hypertensive rats. *JPN Circ. J.* 27 282–293. 10.1253/jcj.27.282 13939773

[B31] Pietranera L. Correa J. Brocca M. E. Roig P. Lima A. Di Giorgio N. (2016). Selective Oestrogen Receptor Agonists Rescued Hippocampus Parameters in Male Spontaneously Hypertensive Rats. *J. Neuroendocrinol.* 28:145. 10.1111/jne.12415 27517478

[B32] Raz N. Lindenberger U. Rodrigue K. M. Kennedy K. M. Head D. Williamson A. (2005). Regional brain changes in aging healthy adults: general trends, individual differences and modifiers. *Cereb. Cortex* 15 1676–1689. 10.1093/cercor/bhi044 15703252

[B33] Sabisz A. Naumczyk P. Marcinkowska A. Graff B. Gasecki D. Glinska A. (2019). Aging and Hypertension - Independent or Intertwined White Matter Impairing Factors? Insights From the Quantitative Diffusion Tensor Imaging. *Front. Aging Neurosci.* 11:35. 10.3389/fnagi.2019.00035 30837864 PMC6389787

[B34] Shaul M. E. Hallacoglu B. Sassaroli A. Shukitt-Hale B. Fantini S. Rosenberg I. H. (2014). Cerebral blood volume and vasodilation are independently diminished by aging and hypertension: a near infrared spectroscopy study. *J. Alzheimers Dis.* 42 S189–S198. 10.3233/JAD-132504 24946871

[B35] Shi H. K. Guo H. C. Liu H. Y. Zhang Z. L. Hu M. Y. Zhang Y. (2020). Cannabinoid type 2 receptor agonist JWH133 decreases blood pressure of spontaneously hypertensive rats through relieving inflammation in the rostral ventrolateral medulla of the brain. *J. Hypertens* 38 886–895. 10.1097/HJH.0000000000002342 32238784

[B36] Strassburger T. L. Lee H. C. Daly E. M. Szczepanik J. Krasuski J. S. Mentis M. J. (1997). Interactive effects of age and hypertension on volumes of brain structures. *Stroke* 28 1410–1417. 10.1161/01.str.28.7.1410 9227693

[B37] Suvila K. Lima J. A. C. Yano Y. Tan Z. S. Cheng S. Niiranen T. J. (2021). Early-but Not Late-Onset Hypertension Is Related to Midlife Cognitive Function. *Hypertension* 77 972–979. 10.1161/HYPERTENSIONAHA.120.16556 33461314 PMC7878356

[B38] Suzuki H. Sumiyoshi A. Matsumoto Y. Duffy B. A. Yoshikawa T. Lythgoe M. F. (2015). Structural abnormality of the hippocampus associated with depressive symptoms in heart failure rats. *Neuroimage* 105 84–92. 10.1016/j.neuroimage.2014.10.040 25462699

[B39] Triantafyllou A. Ferreira J. P. Kobayashi M. Micard E. Xie Y. Kearney-Schwartz A. (2020). Longer Duration of Hypertension and MRI Microvascular Brain Alterations Are Associated with Lower Hippocampal Volumes in Older Individuals with Hypertension. *J. Alzheimers Dis.* 74 227–235. 10.3233/JAD-190842 32039844 PMC7175941

[B40] Van Etten E. J. Bharadwaj P. K. Nguyen L. A. Hishaw G. A. Trouard T. P. Alexander G. E. (2020). Right hippocampal volume mediation of subjective memory complaints differs by hypertension status in healthy aging. *Neurobiol. Aging* 94 271–280. 10.1016/j.neurobiolaging.2020.06.012 32688134 PMC9119497

[B41] Wang Y. Zhang R. Tao C. Xu Z. Chen W. Wang C. (2018). Blood-Brain Barrier Disruption and Perivascular Beta-Amyloid Accumulation in the Brain of Aged Rats with Spontaneous Hypertension: Evaluation with Dynamic Contrast-Enhanced Magnetic Resonance Imaging. *Korean J. Radiol.* 19 498–507. 10.3348/kjr.2018.19.3.498 29713228 PMC5904477

[B42] Whelton P. K. Carey R. M. Aronow W. S. Casey D. E. Collins K. J. Dennison Himmelfarb C. (2018). 2017 ACC/AHA/AAPA/ABC/ACPM/AGS/APhA/ASH/ASPC/NMA/PCNA Guideline for the Prevention, Detection, Evaluation, and Management of High Blood Pressure in Adults: A Report of the American College of Cardiology/American Heart Association Task Force on Clinical Practice Guidelines. *Hypertension* 71 13–115 e. 10.1161/HYP.0000000000000065 29133356

[B43] Willeman M. N. Chawla M. K. Zempare M. A. Biwer L. A. Hoang L. T. Uprety A. R. (2019). Gradual hypertension induction in middle-aged Cyp1a1-Ren2 transgenic rats produces significant impairments in spatial learning. *Physiol. Rep.* 7:e14010. 10.14814/phy2.14010 30916484 PMC6436186

