# Supplementary material for: Evolution of Brain Morphology in Spontaneously Hypertensive and Wistar-Kyoto Rats From Early Adulthood to Aging: A Longitudinal Magnetic Resonance Imaging Study
Source: Front Aging Neurosci. 2021 Nov 30;13:757808. doi: 10.3389/fnagi.2021.757808 (PMC8670306; doi:10.3389/fnagi.2021.757808)
Supplement: Supplementary file 1 [file Data_Sheet_1.docx]

Supplementary Material

# Supplementary Tables

**Supplementary Table 1**

The body weights (g) of the SHRs and WKY rats at 10, 24, 52, and 80 weeks old. The body weight was higher in SHRs than in WKY rats at 52 weeks old, while there was no difference between groups at other time points.

| Group | 10 weeks | 24 weeks | 52 weeks | 80 weeks |
| --- | --- | --- | --- | --- |
| SHR | 222±12 | 345±20 | 399±15 | 337±19 |
| WKY | 225±7 | 326±27 | 358±27 | 353±17 |

**Supplementary Table 2**

The smoothing kernel was the only arbitrary variable in the data processing procedure. A 4 mm full width at half maximum Gaussian kernel was used to smooth the modulated gray matter volume images. Gaussian smooth kernels of 2 mm, 3 mm, 4 mm, 6 mm, and 8 mm were used to smooth the modulated gray matter volume images. Voxel-based morphometry with different Gaussian smooth kernels about hypertension interacting with aging on the gray matter are listed below (Familywise error, p < 0.05; threshold of 200 voxels). Space smoothing can enhance the statistical effect; however, an excessively large Gaussian smooth kernel will hide some significant brain regions. For example, the S1FL and S1BF (right) are buried in the caudate putamen with an 8 mm Gaussian smooth kernel.

| Brain regions | 2 mm | 3 mm | 4 mm | 6 mm | 8 mm |
| --- | --- | --- | --- | --- | --- |
| Septal region | √ | √ | √ | √ | √ |
| Caudate putamen L | √ | √ | √ | √ | √ |
| Caudate putamen R | √ | √ | √ | √ | √ |
| Hippocampus L |  | √ | √ |  |  |
| Hippocampus R |  | √ | √ | √ | √ |
| S1FL L | √ | √ | √ | √ | √ |
| S1BF R, S1FL R | √ | √ | √ | √ |  |
| Cerebellum |  | √ | √ | √ | √ |
| Periaqueductal gray |  |  | √ | √ | √ |
| Caudate putamen L |  |  | √ | √ | √ |
| Caudate putamen R | √ | √ | √ | √ | √ |
| Accumbens nucleus R | √ | √ | √ | √ | √ |
| Thalamus R |  |  | √ | √ | √ |
| Cingulate cortex area 2 R, L | √ |  |  |  |  |
| Dorsolateral entorhinal cortex R |  |  |  | √ | √ |
| Hypothalamic area |  |  |  | √ | √ |
| Olfactory bulb R |  | √ |  |  |  |
| Amygdaloid R |  |  |  | √ | √ |

L, left; R, right; S1FL, Primary somatosensory cortex forelimb region; S1BF, Primary somatosensory cortex barrel field. √ indicates hypertension interacting with aging on the gray matter volume in this region.

**Supplementary Table 3**

The lateral differences in the volume were statistically significant at 4 time points using paired t-tests. There was no bilateral difference in caudate putamen volume in either group at any time point, while the volume of other selected regions significantly differed between hemispheres at certain time points.

| Brain regions | Group | 10 weeks | | 24 weeks | | 52 weeks | | 80 weeks | |
| --- | --- | --- | --- | --- | --- | --- | --- | --- | --- |
|  |  | t | p | t | p | t | p | t | p |
| Caudate putamen | SHR | 0.702 | 0.505 | 1.396 | 0.205 | 0.412 | 0.693 | 1.396 | 0.206 |
|  | WKY | 0.757 | 0.491 | 0.251 | 0.814 | 2.168 | 0.096 | 0.543 | 0.616 |
| Hippocampus | SHR | 6.220 | 0.000 | 0.303 | 0.771 | 1.979 | 0.088 | 1.953 | 0.092 |
|  | WKY | 0.404 | 0.707 | 1.402 | 0.234 | 1.086 | 0.338 | 1.543 | 0.198 |
| Accumbens nucleus | SHR | 10.427 | 0.000 | 7.911 | 0.000 | 5.229 | 0.001 | 16.908 | 0.000 |
|  | WKY | 17.746 | 0.000 | 6.366 | 0.003 | 4.053 | 0.015 | 6.487 | 0.003 |
| Amygdaloid | SHR | 11.019 | 0.000 | 2.612 | 0.035 | 0.165 | 0.873 | 1.217 | 0.263 |
|  | WKY | 4.760 | 0.009 | 2.411 | 0.073 | 0.638 | 0.558 | 1.749 | 0.155 |
| Olfactory bulb | SHR | 1.887 | 0.101 | 0.896 | 0.400 | 0.968 | 0.365 | 1.396 | 0.205 |
|  | WKY | 0.838 | 0.449 | 0.252 | 0.813 | 0.907 | 0.416 | 0.517 | 0.632 |
| Cingulate cortex area 2 | SHR | 1.427 | 0.197 | 1.418 | 0.199 | 1.397 | 0.205 | 0.660 | 0.530 |
|  | WKY | 1.657 | 0.173 | 2.175 | 0.095 | 4.829 | 0.008 | 0.582 | 0.592 |
| S1FL | SHR | 2.685 | 0.031 | 0.541 | 0.605 | 1.198 | 0.270 | 2.444 | 0.044 |
|  | WKY | 2.405 | 0.074 | 1.261 | 0.276 | 4.951 | 0.008 | 0.873 | 0.432 |
| S1BF | SHR | 1.369 | 0.213 | 6.230 | 0.000 | 5.970 | 0.001 | 1.049 | 0.329 |
|  | WKY | 2.000 | 0.116 | 2.631 | 0.058 | 0.775 | 0.482 | 8.323 | 0.001 |
| Primary motor cortex | SHR | 3.803 | 0.007 | 0.180 | 0.862 | 0.500 | 0.633 | 0.497 | 0.634 |
|  | WKY | 1.749 | 0.155 | 0.022 | 0.983 | 0.889 | 0.424 | 1.101 | 0.333 |

# Supplementary Figures

**Supplementary Figure 1.**

The variation in body weight of spontaneously hypertensive rats (SHRs) and Wistar-Kyoto (WKY) rats with time (A). The systolic blood pressure (SBP), diastolic blood pressure (DBP), and mean blood pressure (MBP) in SHRs were higher than in WKY rats at 20 weeks old (B).

**Supplementary Figure 2.**

The signal-to-noise (SNR) of the original T2-weighted images in caudate putamen (CPU), hippocampus (HIP), parietal lobe (Par) and corpus callosum (CC)

**
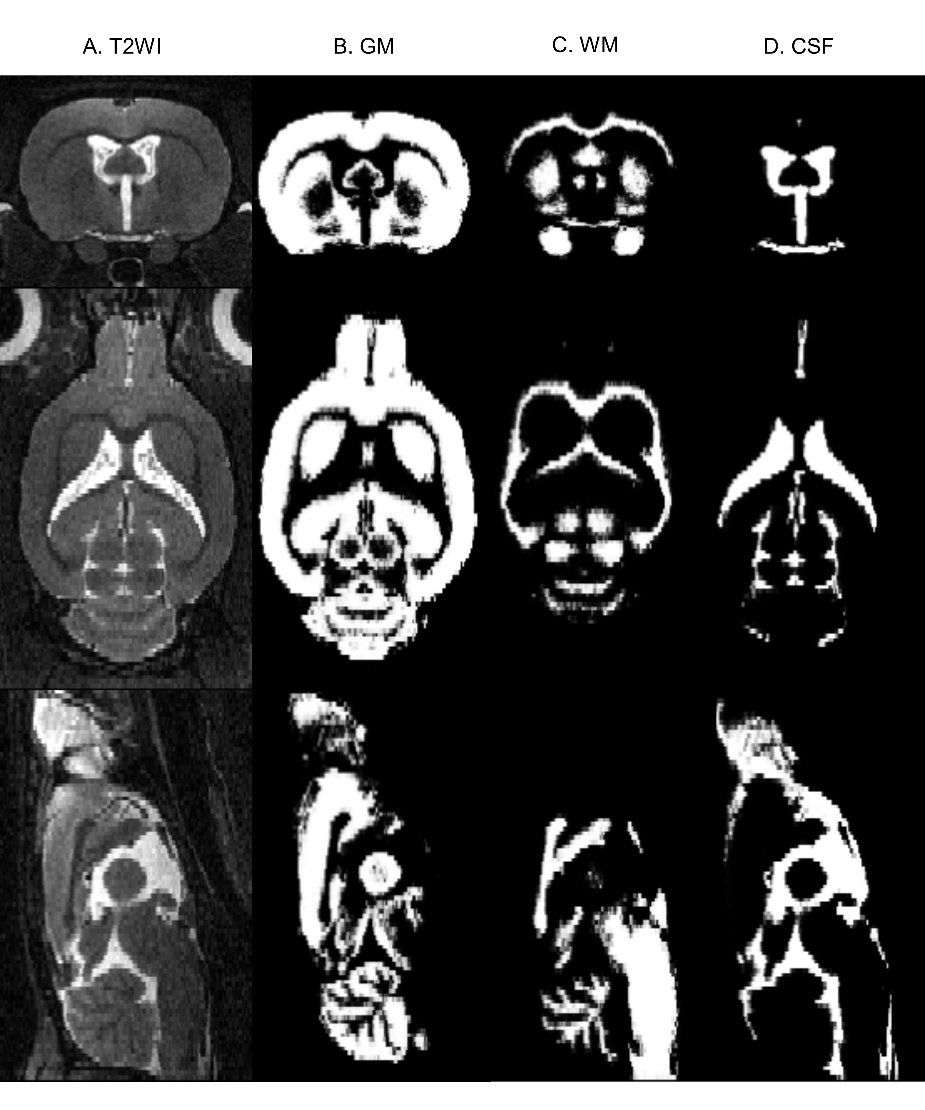
**

**Supplementary Figure 3.**

Coronal, axial and sagittal slices of the original T2-weighted images and individual segmented GM, WM, and CSF images for the SHR brain. A. T2-weighted images (T2WI). B. Gray matter images (GM). C. White matter images (WM). D. Cerebrospinal fluid images (CSF).


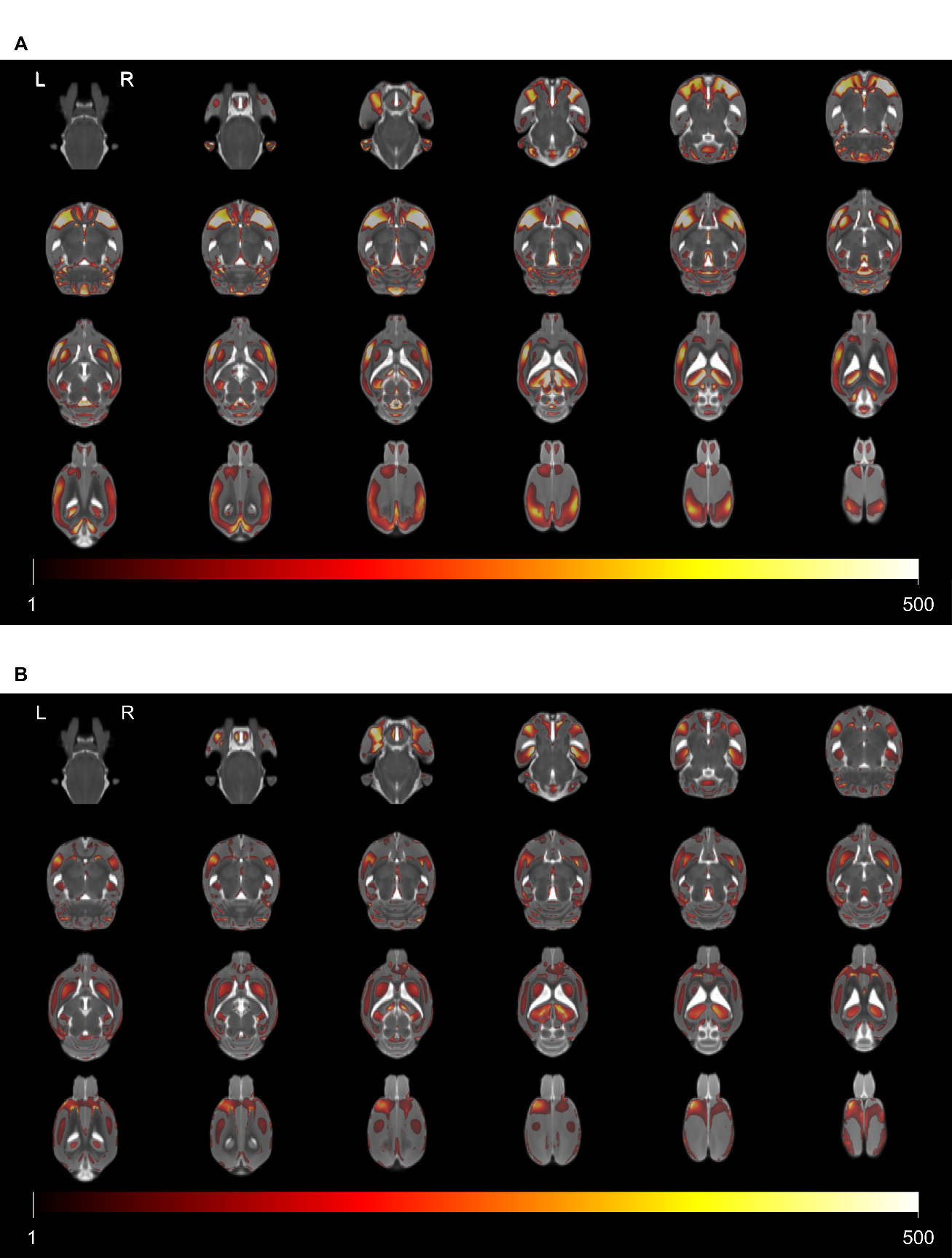


**Supplementary Figure 4.**

Comparisons of main effect of hypertension (A) and age (B) on gray matter volume in SHRs and WKY rats from adulthood to old age. Hypertension has a greater impact on gray matter volume than age.

**Supplementary Figure 5.**

Comparisons of gray matter volume longitudinal changes in SHRs and WKY rats aged 10, 24, 52, and 80 weeks using the region of interest-based method. There were no interactive effects of group and age in these regions.
